# Trapping of a Single Microparticle Using AC Dielectrophoresis Forces in a Microfluidic Chip

**DOI:** 10.3390/mi14010159

**Published:** 2023-01-08

**Authors:** Yanjuan Wang, Ning Tong, Fengqi Li, Kai Zhao, Deguang Wang, Yijie Niu, Fengqiang Xu, Jiale Cheng, Junsheng Wang

**Affiliations:** 1 Software Institute, Dalian Jiaotong University, Dalian 116028, China; 2 Liaoning Key Laboratory of Marine Sensing and Intelligent Detection, College of Information Science and Technology, Dalian Maritime University, Dalian 116026, China; 3 College of Information Science and Technology, Dalian Maritime University, Dalian 116026, China

**Keywords:** trap, dielectrophoresis, microfluidic chip, manipulation

## Abstract

Precise trap and manipulation of individual cells is a prerequisite for single-cell analysis, which has a wide range of applications in biology, chemistry, medicine, and materials. Herein, a microfluidic trapping system with a 3D electrode based on AC dielectrophoresis (DEP) technology is proposed, which can achieve the precise trapping and release of specific microparticles. The 3D electrode consists of four rectangular stereoscopic electrodes with an acute angle near the trapping chamber. It is made of Ag–PDMS material, and is the same height as the channel, which ensures the uniform DEP force will be received in the whole channel space, ensuring a better trapping effect can be achieved. The numerical simulation was conducted in terms of electrode height, angle, and channel width. Based on the simulation results, an optimal chip structure was obtained. Then, the polystyrene particles with different diameters were used as the samples to verify the effectiveness of the designed trapping system. The findings of this research will contribute to the application of cell trapping and manipulation, as well as single-cell analysis.

## 1. Introduction

The precise trapping and manipulation of individual cells in three dimensions provide many possible applications in regenerative medicine, tissue engineering, neuroscience, and biophysics [1]. For example, cellular super-resolution imaging techniques place stringent requirements on sample preparation, and the target cells must be immobilized for a period to achieve high-precision imaging [2]. Due to the high heterogeneity of cells, it is important to assay individual cells, and single-cell analysis reveals significant physiological characteristics of individual cells, such as metabolism, protein levels, and gene expression [3,4,5,6]. Biomolecular analysis at the single-cell level can isolate subpopulations that are resistant to chemotherapy or at higher risk of metastasis from heterogeneous tumor cells [7]. In the real-time monitoring of single-cell dynamics, researchers and industry require knowledge of the definitive status of cell phenotypes during each stage of expansion, maturation, or differentiation. For example, to reveal the impact of the initial phenotype of stem cells on subsequent fates, including proliferation, self-renewal, differentiation, and apoptosis, and to study their ultimate therapeutic potential, it is necessary to conduct studies at the single-cell level [8,9,10]. Therefore, capturing and releasing particles or cells in a controlled manner, as well as precisely capturing and manipulating single or countable cells in a test area is a prerequisite for cell analysis [11].

Microfluidic technology has the advantages of lower sample consumption, short reaction time, flexible design, high throughput, and easy integration, which makes it widely used in biomedicine, cell analysis, environmental detection, food safety, and other fields. Single-cell isolation, capture, transport, and processing can be easily and precisely performed using the microfluidic platform. In addition, microfluidic chips can carry out precise fluid control and have good scale compatibility between microchannels and cells, so they are uniquely suited to control and manipulate individual cells [12,13,14,15].

Commonly used microparticle capture technologies based on the microfluidic chip include optics, magnetism, acoustics, hydrodynamics, and electricity [16,17]. Among them, hydrodynamic capture is independent of external forces, and the capture is achieved by designing special channel structures to control the field-flow distribution and thus restrict the movement of microparticles. It has received a lot of attention because of its simplicity, low cost, ease of integration, and harmlessness [18,19]. High-throughput cell capture can be achieved by designing complex channel structures [20], but complex channel structures are prone to clogging. Tanyeri [21] et al. ingeniously used the stagnant flow generated by the microfluidic device to achieve the trap of microscale and nanoscale particles. Shenoy [22] et al. developed a Stokes trap that achieved the precise manipulation of multiple particles and fluidic-directed assembly of multiple particles, but precise control of the fluid is required.

Optical methods use optical traps to manipulate single cells. For example, an optical tweezer is a single-beam optical gradient trap, which can accurately trap and rotate single cells by manipulating the cells toward the focus of the laser beam by optical force [23,24]. However, optical tweezer-based cell manipulation is usually accompanied by costly optics and complex manipulation procedures and may cause physiological damage to cells and biological samples due to laser-induced heating; more importantly, optical tweezers are not competent at high-throughput capture. Magnetic capture enables the capture and manipulation of cells by combining them with nanoscale immunomagnetic beads. This method has high specificity and is a commonly used method for cell trapping [25,26]. However, this method requires the pretreatment of cells to bind magnetic nanoparticles to targeted cells, which is complicated and costly and may cause damage to the biological activity of the cells.

Acoustic technology is a commonly used method for label-free manipulation: a surface acoustic wave (SAW) is generated by giving alternate electric current signals at a resonance frequency to an interdigitated transducer (IDT) deposited on a piezoelectric substrate. SAW can focus ultrasonic energy on specific points and generate acoustic nodes and antinodes for the noninvasive manipulation of micron and nanoparticle particles. Daniel Ahmed [27] et al. described an acoustic-based, on-chip manipulation method, and the precise rotation of *C. elegans* was achieved by driving microbubbles to produce stable vortices. Similarly, Lubli N. F. [28] et al. introduced a bubble-based acoustic streaming microfluidic device that could achieve the controlled and reliable out-of-plane rotation of single *HeLa* cells. There have also been numerous reports of cellular manipulation using acoustic forces [29,30], due to the low cost, being label-free and little damage to cells; however, acoustic-based devices have drawbacks for the manipulation of cells and biomolecules, such as an essential need for a bulky function generator and amplifier that limit the portability of the SAW-based platforms [31].

The electrical method has been widely considered because of its convenience, low cost, and controllability. One of the most compelling electrical methods is the DEP technique, which relies on the dielectric properties of the particles and allows for highly selective and sensitive analysis [32]. Precise particle control can be achieved by designing unique electrode structures and applying signals, with the advantages of being label-free, contactless, using a lower sample consumption, fast, and easy to integrate. DEP technology has proven to be a versatile mechanism for manipulating various microfluidic/nanoscale biological particles (i.e., cells, viruses, bacteria, DNA, and proteins) [33]. Back in 2002, Professor J. Voldman of MIT designed a quadrupole electrode array to trap and sort cells [34]. They established a prediction model and a set of design rules to optimize the trap [35,36]. Later, many studies were reported using DEP technology to achieve the capture of particles or cells, for example, Guo et al. [37] designed a graphic array consisting of multiple quadrupole electrode units, with a pair of central electrodes in the center of each quadrupole electrode unit. Live cells were captured using nDEP forces and were moved in a controlled manner. Jaione et al. [38] proposed an efficient and reversible mechanism to capture microbeads by a combination of DEP and mechanical traps, which allows multiple manipulations of microbeads by DEP technology. Kim et al. [39] designed an advanced electroactive double-well array for deterministic single-cell capture and on-chip analysis using DEP and reaction wells. Alicia M. et al. [40] used the polarization effect of the Janus sphere to transform it into a mobile floating electrode in the electric field, which can selectively capture and release targets for cargo transport. Meenal Goel et al. [41] used surface pattern electrodes to drive and control live pathogenic *Salmonella typhimurium* and *Escherichia coli (E. coli*) based on AC dielectrophoresis (AC-DEP). Chen et al. [42] successfully captured single cells and localized them in pore arrays by integrating DEP capture techniques with pore arrays of different pore sizes. The above studies were focused on multicellular capture and control using electrode arrays [43], but there are few studies on the reversible capture and manipulation of single cells or particles. Prateek Benha et al. [44] designed an open electrode structure consisting of four wall electrodes for the rotation of bovine oocytes. However, the method involves placing the bovine oocytes into the chamber using a mouth pipette, the structure can only manipulate larger cells, and the open structure is susceptible to contamination. Habaza et al. [45] used the DEP technique to capture and accurately rotate cells and achieved the 3D imaging of cancer cells and leukocytes, but the structure of this method was complex. Chen et al. [46] integrated fluid manipulation, cell capture, and single-cell impedance analysis into a parallel-plate chip. *HeLa* cells were manipulated by liquid electrophoresis (LDEP) and DEP but this method requires complex liquid driving. In addition, some scholars are devoted to the study of the dynamic model of particle trajectory under the time-varying force field and optimize the device parameters for better trapping [47,48].

Inspired by the previous studies, this paper designed a unique 3D quadrupole electrode structure based on AC-DEP technology. Different from the traditional ITO planar electrode, the height of the three-dimensional electrode is the same as the channel, which ensures the consistency of the electric field in the direction of the channel height, and the particles can be subjected to DEP forces in the whole range of channel height. The 3D quadrupole electrode structure designed here allows for the easy trapping and release of single cells and does not harm the cells. In addition, numerical simulations of electrode shape, angle, and channel width were carried out, the influence of chip geometry on the trapping effect was analyzed, and the optimized chip structure was designed. In addition, polystyrene particles with different diameters were used as the samples to verify the trapping effect of the designed chip. Those findings will contribute to the capture and manipulation of cells, as well as single-cell analysis.

## 2. Materials and Methods

### 2.1. Theory

DEP refers to the phenomenon of electroneutral particles moving in a nonuniform electric field due to being polarized. When electrically neutral particles are in the electric field, under the action of the electric field force, the free charges in the electrolyte solution and particles will be rearranged, that is, polarized, which will lead to the enrichment of some heterogeneous-induced charges at the interface between particles and solution. In the nonuniform electric field, the positively and negatively induced charges are in different electric field strengths, i.e., they are subjected to different electric field forces, so the combined force on the particles is not zero, and the particles will move under the action of electric field forces. The force on a particle in a nonuniform electric field due to being polarized is called the DEP force.

The conventional DEP force on a homogeneous spherical particle is given by [49]:

(1) FDEP=2πεmr3Re(fcm*(ω))∇E2

where *r* is the particle radius, *E* is the electric field strength,

(2) $$f_{\mathrm{cm}}^{*}(\omega )=\frac{\varepsilon_{p}^{*}-\varepsilon_{m}^{*}}{2\varepsilon_{m}^{*}+\varepsilon_{p}^{*}}$$

*f*_cm_ is the Clausius–Mossotti factor, CM factor for short, *ε*_p_ is the dielectric constant of the particle, and *ε*_m_ is the dielectric constant of the solution. When the degree of polarization of the particles is greater than that of the solution, *ε*_p_* − *ε*_m_* > 0, *f*_cm_ > 0, and under the action of the pDEP force, the particles are attracted to the place where the electric field is relatively strong. On the contrary, when the polarization of the particle is smaller than that of the medium, *ε*_p_* − *ε*_m_* < 0, *f*_cm_ < 0, and the particle is repelled to the place where the electric field strength is relatively weak by the effect of the nDEP force. Therefore, the CM factor determines the direction of the DEP force on the particles.

The CM factor is a complex form:

(3) $$\varepsilon^{*}=\varepsilon -i\frac{\sigma }{\omega }$$

where *ε* is the permittivity, *σ* is the conductivity, and *ω* is the angular frequency of the electric field.

### 2.2. System Design

**Figure 1:**
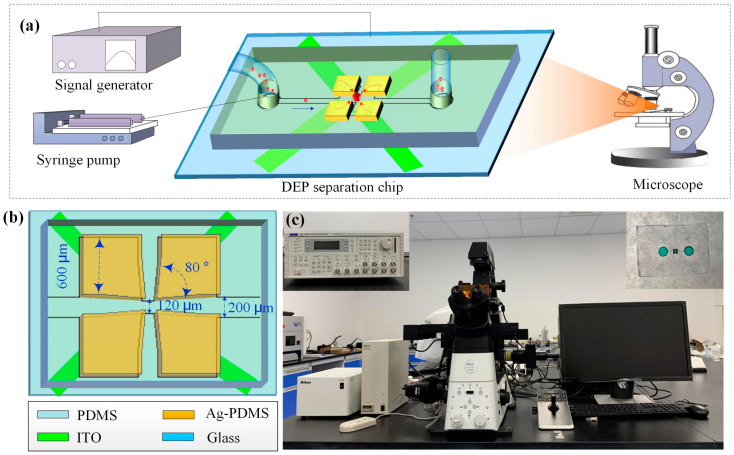
(**a**) Structure of the capture system, (**b**) 3D electrode, and (**c**) photograph of the system.

According to the above theoretical analysis, a microfluidic particle trapping and manipulation system was designed. The whole system consists of a four-phase AC signal generator (AIM-TTI, TGA12104, Huntingdon, UK), microscope (Nikon Eclipse Ti-E, inverted fluorescence microscope, EINST Technology Pte Ltd., Singapore), CCD, precision syringe pump (Longer-LSP10-1B, Longer Precision Pump Co., Ltd., Baoding, China), separation chip and computer. The structure of the capture system is shown in Figure 1a. Among them, the microfluidic particle capture chip is the most important part of the whole system, as shown in Figure, which consists of four layers, from bottom to top, a glass substrate, an ITO wire layer, a 3D three-dimensional electrode, and a PDMS channel. An inlet well was designed on the left side of the main channel, the particle trapping chamber was in the middle, and an outlet well was designed on the right side. The width of the main channel is 200 μm. The height of the passage is 38 μm. The electrode is a 3D structure with the same height as the channel. It is composed of four rectangular electrodes with a side length of 600 μm, and the angle near the trapping chamber is 80°. The electrode is made of a mixture of PDMS gel (Dow Corning, Midland, MI, USA) and Ag powder with a diameter of 1~3 μm (Xingrongyuan, Beijing, China) at a weight ratio of 15:85. Ag–PDMS has the dual properties of Ag and PDMS. It has good electrical conductivity and easily bonds with PDMS material, which ensures the tightness of the channel and avoids the leakage of fluid. The ITO layer acts as a wire connecting the stereoscopic electrode and the signal generator. The unique 3D electrode structure designed here can generate a strong electric field intensity gradient in the whole capture chamber, ensuring the trapping effect. Figure 1c shows a photograph of the system. See the Supplementary Materials for fabrication details of the chip [50].

### 2.3. Sample Preparation

#### 2.3.1. The Preparation of Phosphate Buffered Saline (PBS) Solution

For DEP technology, the conductivity of the buffer is an important factor affecting the dielectric response of particles. PBS is a commonly used buffer solution, which can easily adjust the conductivity of the solution and can maintain the activity of biological particles.

PBS solution was prepared by placing 100 mL of deionized water into a beaker, PBS solution was added dropwise with a pipette, and the conductivity of the solution was measured using a conductivity tester until the conductivity of the solution was 3 mS/m.

#### 2.3.2. The Preparation of Polystyrene Particles

Polystyrene particles with diameters of 20 μm and 30 μm were used as the samples. First, a pipette was used to drop a few drops of polystyrene particles into a 1.5 mL tube containing the prepared PBS solution and was shaken evenly. Then, the tubes were placed in a centrifuge at 8000 rpm and centrifuged for 5 min at room temperature. The supernatant in the tubes was then removed and the prepared PBS solution was added again and shaken well. Centrifugation was repeated twice, then the polystyrene particle suspension with a solution conductivity of 3 mS/m was obtained.

### 2.4. Experimental Steps

First of all, the microfluidic trapping chip was placed in a plasma cleaner for 1 min to increase the hydrophilicity of the channel. Then, the prepared PBS solution was passed into the inlet of the chip to eliminate the bubbles in the channel. Each of the four electrodes of the capture chip was connected to the four-phase AC signal generator. The prepared polystyrene particle suspension was loaded into the syringe pump, the appropriate flow rate was set, and then a hose was used to connect the sample to the chip inlet. The chip was placed on the microscope stage and the focal length was adjusted. The sample was slowly flowed into the capture chip using the syringe pump. When the polystyrene particles reached the capture region, the particles were confined in the trapping chamber due to the DEP force, and the trajectories of the particles under the electric field were recorded by microscope and CCD.

## 3. Results and Discussion

### 3.1. Electrode Structure Optimization and Numerical Simulation

In order to obtain the optimal chip structure, simulations were conducted in terms of electrode shape, electrode angle, and the width of the microchannels. COMSOL Multiphysics 5.4 software was used to simulate the aspects of electric field intensity gradient, electric field, and field flow. The laminar flow module and AC/DC module of the COMSOL Multiphysics software were used to simulate the field flow, electric field, and electric field intensity gradient of the chip. Firstly, the electrode model was imported into the COMSOL Multiphysics software. Then, the material was defined and the boundary conditions of the current were set. The channel wall was defined as insulation. The two groups of electrodes on the diagonal were set as +10 V and −10 V. The optimized electrode structure was obtained according to the simulation results.

#### 3.1.1. The Influence of Electrode Shape

**Figure 2:**
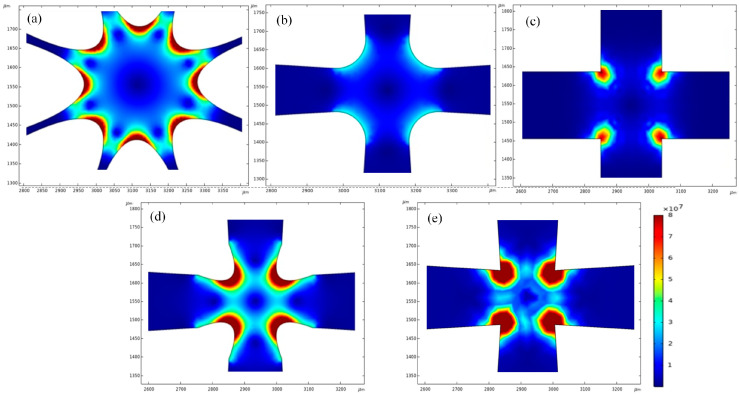
The simulation of electric field intensity gradient of electrodes with different shapes (V^2^/m^3^); (**a**) octupole elliptical structure, (**b**) quadrupole circular structure, (**c**) quadrupole right-angle structure, (**d**) quadrupole elliptical structure, and (**e**) quadrupole sharp-angle structure.

In this paper, the distribution of electric field intensity gradients of common electrode shapes such as ellipse, circular, right angle, or sharp angle were simulated. By setting the channel width as 200 μm, the electric field intensity gradient generated by electrodes of different shapes was numerically simulated, and the simulation results are shown in Figure 2.

The electrode of Figure 2a is an octupole elliptical structure. From the simulation results, it can be seen that the distribution of the electric field intensity gradient between electrodes is continuous, but the action range is relatively small, only the elliptic tip has a strong electric field intensity gradient. In addition, the proximity between electrodes leads to a narrow channel for particle flow. Figure 2b shows a quadrupole circular structure, which can provide a wider channel for particle flow; however, the electric field intensity gradient of this electrode is significantly weaker than the other structures. Figure 2c shows a quadrupole right-angle structure. According to the simulation results, although the electric field intensity gradient of this structure is stronger than that of Figure 2b, its action range is small, which is not conducive for capture. Figure 2d shows a quadrupole elliptic structure. According to the simulation results, the electric field intensity gradient of the structure has a relatively wide distribution range and can generate a strong DEP force. Figure 2e shows a quadrupole sharp-angle structure, and it can be seen from the simulation results that the electric field intensity gradient of this structure is stronger and the action range is even wider than that of Figure 2d. Therefore, according to the simulation results, the quadrupole sharp-angle structure shown in Figure 2e is the best choice. It has a more ideal distribution of electric field intensity gradients, which can generate a wide range and strong DEP force. In addition, it also can provide a wider channel for the flow of the particles.

#### 3.1.2. The Influence of Electrode Angle

**Figure 3:**
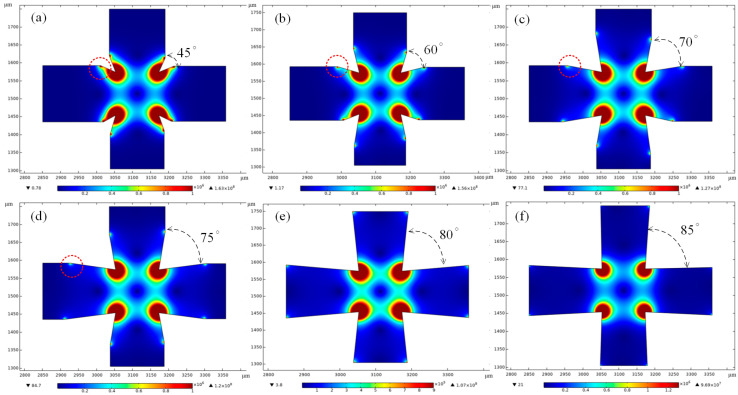
Simulation of electric field intensity gradient of a triangular electrode at different angles (V^2^/m^3^); (**a**) 45°, (**b**) 60°, (**c**) 70°, (**d**) 75°, (**e**) 80°, and (**f**) 85°.

Previous analysis has shown that the acute angle electrode is superior to other shapes. Next, the influence of electrode angle on the electric field intensity gradient was analyzed by numerical simulation. The included angle of the electrode was adjusted while keeping other parameters unchanged, as shown in Figure 3, and from Figure 3a–f, the included angles were 45°, 60°, 70°, 75°, 80°, and 85°, respectively. It can be seen from Figure 3a–d, when the electrode angle is relatively small, there will be a strong DEP force at the inflection point near the trapping chamber (red circle in the figure), which will interfere with the trapping of particles. As for Figure 3f, it can be seen from the simulation result that the range of electric field intensity gradient generated is reduced because the 85° angle is close to a right angle. By comparing the simulation results of electrodes with different angles, it can be seen that the electrode with an angle of 80° has the largest range of electric field intensity gradient, which can cover the entire trapping chamber and with strong intensity. Furthermore, there is no interference caused by an inflection point. Moreover, the electric field intensity in the center of the chamber is the lowest. Therefore, under the action of the DEP force, the particles will be trapped in the center of the chamber and cannot escape. So, 80° is the optimal choice of electrode angle.

#### 3.1.3. The Influence of Channel Width

**Figure 4:**
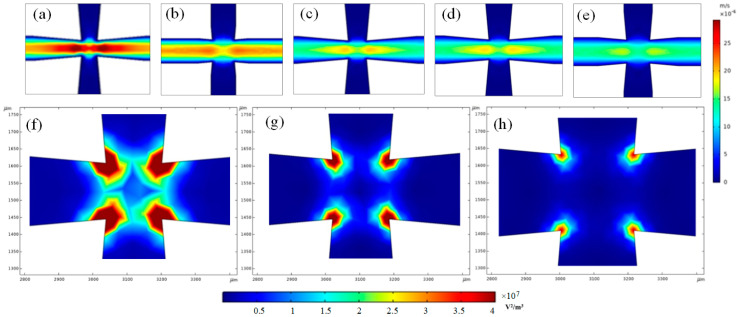
Numerical simulation of different channel widths; (**a**–**e**) represent the simulation of the flow field with channel widths of 100 μm, 150 μm, 200 μm, 250 μm, and 300 μm; (**f**–**h**) represent the simulation of electric field intensity gradient with the channel widths of 200 μm, 250 μm, and 300 μm.

As we know, due to the effect of fluid force, particle capture requires a low flow rate. Under the same initial flow rate, the wider the channel, the lower the flow rate and the less likely it is to block. However, the widening of the channel results in a wide distance between the electrodes, and the electric field force cannot affect the entire chamber. In order to balance the flow rate and the DEP force, five different channel widths of 100 μm, 150 μm, 200 μm, 250 μm, and 300 μm were simulated in terms of the flow rate and the electric field intensity gradient. The simulation results are shown in Figure 4. Figure 4a–e is the flow field simulation of chips with five different channel widths. It can be seen from Figure 4a,b that at the same initial flow rate, when the channel width was 100 μm and 150 μm, due to the narrow channel the flow rate was relatively fast, and it was difficult to achieve particle capture. It can be seen from Figure 4c–e that the flow rates decrease gradually when the channel widths are 200 μm, 250 μm, and 300 μm, which is helpful for particle capture. For the three channel widths, this study also conducted simulation analysis from the aspect of electric field intensity gradient, and the results are shown in Figure 4f–h. As can be seen from Figure 4, as the channel width increases, the distance between the electrodes increases, which results in a corresponding decrease in the generated DEP force. Therefore, according to the simulation results of the flow field and electric field, 200 μm was the optimal channel width.

### 3.2. The Electric Field Simulation under the Optimized Structure

Through the above numerical simulation of electrode shape, angle, and channel width, the optimal electrode structure was obtained. Next, the captured and rotating electric field of the optimized chip structure was simulated and analyzed.

#### 3.2.1. Trapping

**Figure 5:**
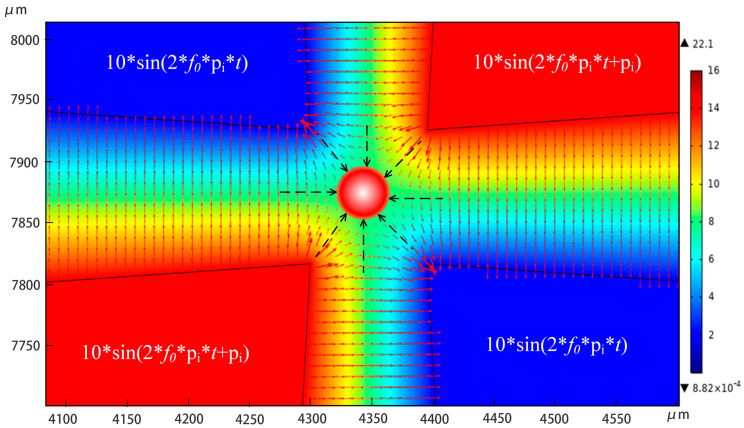
Electric field intensity simulation of particle capture (V/m).

To achieve particle trapping, the voltages of the four electrodes were set clockwise to 10sin(2*f*_0_π*t* + π), 10sin(2*f*_0_π*t*), 10sin(2*f*_0_π*t* + π), and 10sin(2*f*_0_π*t*), and the frequency *f*_0_ was set to 500 kHz. The electric field simulation results of the particle capture are shown in Figure 5. As can be seen from the figure, the sharp corner of the electrode has the densest electric field lines and the largest electric field intensity, while the center area has the lowest electric field intensity. Therefore, the particles will be trapped in the center of the chamber due to the action of the electric field force pointing from the periphery to the center.

#### 3.2.2. Rotate

**Figure 6:**
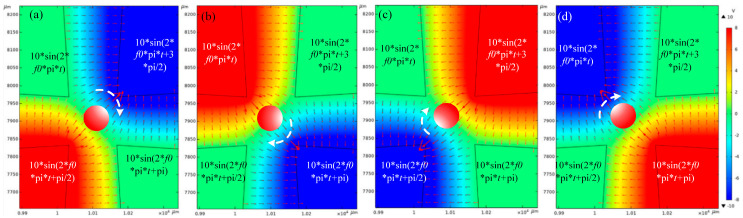
Electric field intensity simulation of particle rotation (V/m); (**a**) 0T moment, (**b**) 1/4T moment, (**c**) 1/2T moment, (**d**) 3/4T moment.

Using the designed electrode structure, four electrodes loaded with π/2 phase difference signals can achieve the rotation of captured particles. The signals of the four electrodes were set clockwise as follows: 10sin(2*f*_0_π*t*), 10sin(2*f*_0_π*t* + 3π/2), 10sin(2*f*_0_π*t* + π), and 10sin(2*f*_0_π*t* + π/2), and the frequency *f*_0_ was set as 200 kHz (To make it easy to calculate the period). The simulation results of the rotating electric field are shown in Figure 6. Figure 6a–d show the situations of 0T, 1/4T, 1/2T, and 3/4T, respectively. It can be seen that under the action of the torque of the rotating electric field, the microparticles will rotate with the change in the direction of the electric field force. Figure 6 shows a complete rotation period and (a)–(d) are the four moments of clockwise rotation of the particle.

### 3.3. The Trapping of Polystyrene Particles

In order to verify the trapping effect of the designed chip, polystyrene particles were used as the samples. The prepared polystyrene particle solution with a conductivity of 3 mS/m was loaded onto the syringe pump and connected to the inlet of the chip through a hose. The chip was fixed on the microscope stage and the focus was adjusted. Wires were used to connect the four outputs of the signal generator to the four electrodes of the chip. The flow rates of polystyrene particles were controlled by the precision syringe pump. When the polystyrene particles flow near the trapping chamber of the microfluidic chip, electrical signals were applied. The capture process of polystyrene particles was recorded by the microscope.

#### 3.3.1. The Trapping of Polystyrene Particles with a Diameter of 30 μm

**Figure 7:**
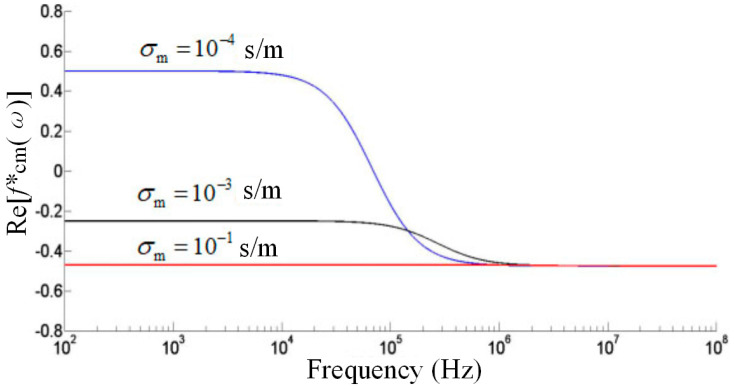
CM factor curve of the polystyrene particles.

In order to obtain the optimal trapping voltage and frequency, we plotted the CM factor curve of the polystyrene particles. As shown in Figure 7, when the solution conductivity was 10^−4^ S/m, the crossing frequency was 70 kHz, and the polystyrene particles were subjected to pDEP forces below 70 kHz and nDEP forces above 70 kHz. When the solution conductivity was 10^−3^ S/m, the polystyrene particles were subjected to an nDEP force within the whole frequency range. When the frequency was 10^5^ Hz, the subjected force was enhanced with the increase in frequency. When the frequency was 10^6^ Hz, the force reached the maximum. Since the solution would cause a disturbance at a higher frequency, 500 kHz was selected as the operating frequency (The conductivity of the solution used here was 3 × 10^−3^ S/m).

**Figure 8:**
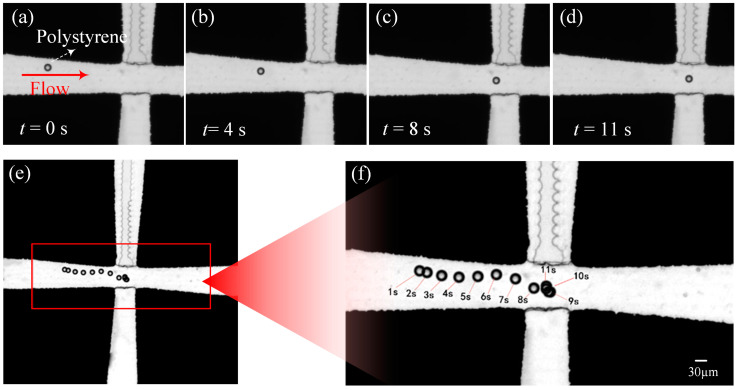
A 30 μm polystyrene particle trapping experiment; (**a**) at 0 s, (**b**) at 4 s, (**c**) at 8 s, (**d**) at 11 s, (**e**) overlay result of the video sequence, and (**f**) the enlargement of (**e**).

Figure 8 shows the trapping process of polystyrene particles with a diameter of 30 μm. The voltage of the applied AC signal was 10 V and the frequency was 500 kHz, the flow rate was 1.5 mm/min. Because of the large particle diameter, the particle can be subjected to strong DEP forces, assuming that the particle flows near the tip of the electrode at the 0 moment, as shown in Figure 8a. From the experimental results, it was found that at the 11th second, as shown in Figure 8d, the particle was successfully trapped in the center of the trapping chamber under the action of the DEP force. As can be seen from the figure, particles enter the trapping chamber at the 8th second. At the 9th second, particles continue to flow along the direction of fluid flow due to the action of fluid force. However, due to the strong DEP force generated by the quadrupole electrode pointing to the chamber center, in the 10th second, the particles returned to the trapping chamber and were trapped in the center of the chamber.

#### 3.3.2. The Trapping of Polystyrene Particles with a Diameter of 20 μm

**Figure 9:**
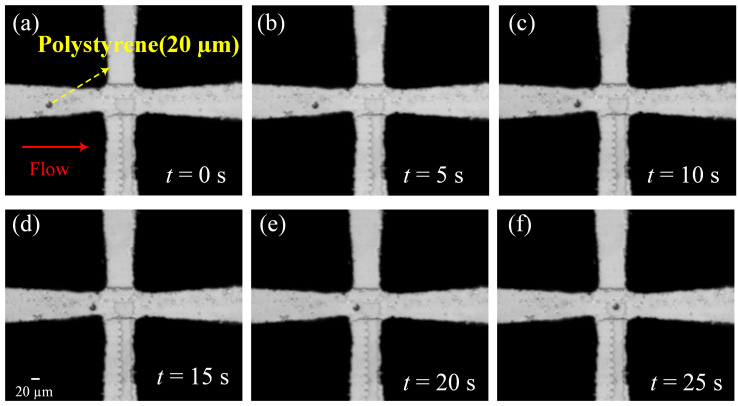
A 20 μm polystyrene particle trapping experiment; (**a**) at 0 s, (**b**) at 5 s, (**c**) at 10 s, (**d**) at 15 s, (**e**) at 20 s, and (**f**) at 25 s.

According to Formula (1), the electrophoretic force on the particle is proportional to the cube of the particle radius. Therefore, the smaller the particle radius, the smaller the DEP force on the particle, and the more difficult it is to trap the particle. In order to verify the effect of particle size on trapping, polystyrene particles with a diameter of 20 μm were used. The amplitude of the external AC signal was 10 V and the frequency was 500 kHz, and the flow rate was 0.6 mm/min. The experimental results are shown in Figure 9. Figure 9a–f shows the trapping of polystyrene particles with a diameter of 20 μm. As can be seen, due to the smaller particle size, a slower flow rate is required in order to overcome the effects of fluid force. We assume that the particle flows near the tip of the electrode at time 0 (Figure 9a, the same position as the 30 μm particle). It takes significantly longer to capture 20 μm particles than 30 μm particles, and the 20 μm particle was not trapped in the chamber until the 25th second.

## 4. Conclusions

Herein, a quadrupole particle trapping system was designed based on AC-DEP technology. The trapping chip contains a central symmetric quadrupole 3D electrode, which is composed of four rectangular electrodes with an angle of 80°. The electrode height is 38 μm, which is the same as the channel height. The stereoscopic electrode can generate a strong and continuous DEP force in the whole channel height space to improve the trapping effect. In this paper, COMSOL software was used to simulate the electrode shape, angle, and width of the channel, and the optimized electrode structure was designed. Polystyrene particles of 30 μm and 20 μm were used as samples to verify the effectiveness of the designed chip. It was found that compared with the polystyrene particles with a diameter of 30 μm, the particles with a diameter of 20 μm suffered weaker DEP forces and needed a slower flow rate to achieve trapping, and the trapping time was significantly longer than that of the 30 μm particles. By adjusting the control parameters, the trapping system proposed here can be conveniently applied to trap other particles, such as polymers, cells, etc. In addition, the trapping chip designed here can also realize the rotation operation of the particles by applying an AC signal with a phase difference of π/2 to the four electrodes. The results of this study will contribute to single-cell trapping and manipulation, as well as single-cell processing and analysis.

## Data Availability

The data that support the findings of this study are available on request from the corresponding author, (J.W.), upon reasonable request.

## Supplementary Materials

The following supporting information can be downloaded at: https://www.mdpi.com/article/10.3390/mi14010159/s1.
