# Supplementary material for: Trapping of a Single Microparticle Using AC Dielectrophoresis Forces in a Microfluidic Chip
Source: Micromachines (Basel). 2023 Jan 8;14(1):159. doi: 10.3390/mi14010159 (PMC9863554; doi:10.3390/mi14010159)
Supplement: Supplementary file 1 [file micromachines-14-00159-s001.zip › micromachines-2063598-supplementary.pdf]

(Supplementary materials)

Trapping of single microparticle based on AC dielectrophresis force in a microfluidic chip

Yanjuan Wang[a,b], Ning Tong[a]， Fengqi Li[a], Kai Zhao[b,c], Deguang Wang[a], Yijie Niu[a], Fengqiang Xu[a], Jiale Cheng[b,c] and Junsheng Wang[b,c,*]

a Software Institute, Dalian Jiaotong University, Dalian 116028, China

b Liaoning Key Laboratory of Marine Sensing and Intelligent Detection, College of Information Science and Technology, Dalian Maritime University, Dalian 116026, China

c College of Information Science and Technology, Dalian Maritime University, Dalian 116026, China

* Correspondence: wangjsh@dlmu.edu.cn

The fabrication of dielectrophoretic separation chips

1. The procedure of chip fabrication

The fabrication process of the separation chip is shown in Figure S1, which includes four steps: (a) The fabrication of ITO wire substrate layer (b) The fabrication of 3D microelectrode layer (c) The fabrication of PDMS channel layer (d) The bonding of DEP separation chip.

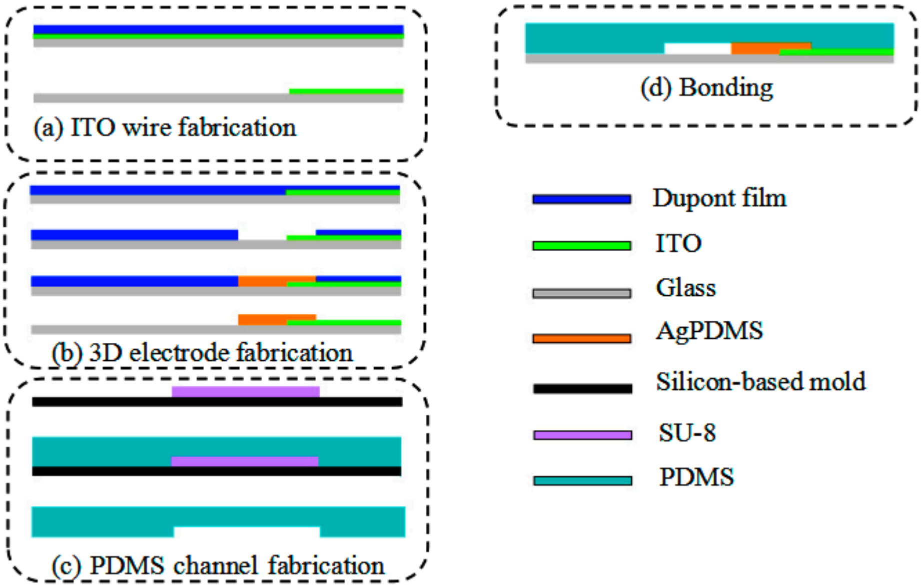

Figure S1. The fabrication process of DEP separation chip (**a**)The fabrication of ITO glass substrate; (**b**) The fabrication of microelectrode; (**c**) The fabrication of PDMS; (**d**) The bonding of the chip

2. The fabrication of ITO wire substrate layer

The ITO conductive glass was selected here. The resistance of the conductive layer is 5 Ω, and the thickness is 200nm. ITO conductive layer with specific shape was etched by wet etching. First use AutoCAD to design the ITO graphics, and then make a mask.

The production of ITO conductive glass substrate is divided into the following steps:

(a) **Cleaning**: First, take a piece of ITO conductive glass with smooth surface and put it into a plasma cleaner (PDC-30G, Harrick Plasma, Ithaca, NY) for cleaning 5 min. Then, rinse the glass surface with alcohol, acetone and deionized water. Finally, blow dry with nitrogen, and then put it into the oven for drying.

(b) **Lamination**: First, cut the DuPont dry film (SD238) to a suitable size, and remove the inner protective film. Then, use a laminator to press the DuPont dry film on the ITO conductive glass. Finally, put the pressed glass piece in a constant temperature oven (Isotempmodel 280A, Fisher Scientific, Pittsburgh, PA) and bake for 3min.

(c) **Exposure:** Since DuPont dry film is a negative photoresist, it will be cured and denatured when exposed to appropriate ultraviolet light. Firstly, the mask is attached to the surface of DuPont dry film. Then exposed to UV for 15s under the ultraviolet exposure machine (2105C2 Radiation Power Systems Inc.), the mask pattern will be transferred onto DuPont dry film. Then bake in constant temperature oven for 5min to promote dry film curing.

(d) **Development**: Put the ITO substrate glass after exposure into 1wt% Na_2CO_3 development solution 5 min for development. As the DuPont dry film irradiated by UV light has been cured, it can not react with the developer, while the unirradiated portion will chemically

react with the Na_2CO_3 development solution and be washed away by the developer. Then, rinse the developed ITO glass with deionized water, dry it with nitrogen, and bake it in a constant temperature oven for 5min.

(e) **Etching**: First, prepare an etching solution, prepare concentrated nitric acid, concentrated hydrochloric acid and deionized water at a volume ratio of 3:50:50, and heat the etching solution to 40°C. Then, the developed ITO glass is put into the etching solution for 1 min. At this time, the ITO conductive film which is not covered by DuPont dry film will react with the etching solution and be cleaned by the etching solution. Finally rinse with deionized water.

(f) **Demembrane**: Firstly, put the etched ITO glass into 5wt% NaOH solution for 5min to remove the dry DuPont film. Then, rinse with alcohol, acetone and deionized water alternately, dry with nitrogen and bake in constant temperature oven for 3min.

Through the above process, ITO conductive glass substrate is finished, the completed ITO wire substrate is shown in Figure S2.

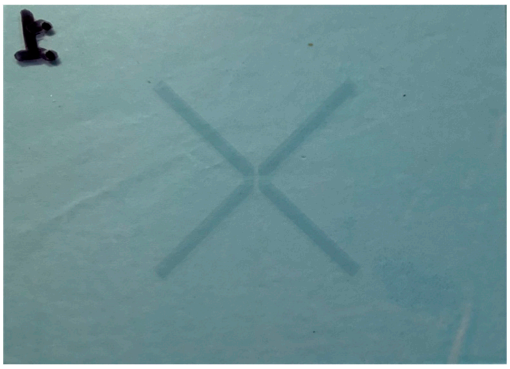

Figure S2. The ITO wire substrate

3. The fabrication of 3D microelectrode layer

Before making a 3D microelectrode, we also need to use AutoCAD software to design the microelectrode graphics, and then make a mask. The electrode mask used here was shown in Figure S3a. The fabrication method of the microelectrode is still using photolithography technology, and the mask pattern is transcribed on the DuPont dry film through ultraviolet exposure. The thickness of DuPont dry film used here is 38 μm, so the height of 3D microelectrode is 38 μm.

The fabrication process of 3D microelectrode is as follows:

(a) **Cleaning**: Same as before.

(b) **Lamination**: Same as before.

(c) **Exposure**: First, attach the mask to the surface of the DuPont dry film. Note that the electrode part of the mask should overlap the ITO wire to ensure that the fabricated microelectrode is connected to the ITO wire. Then put it under the exposure machine, and exposed for 5s, transfer the mask pattern to DuPont dry film, and bake it in constant temperature oven for 3min to strengthen the dry film curing.

(d) **Development**: Put the exposed glass into 1wt% Na_2CO_3 development solution for development, and the development time is 5min. The electrode pattern transferred onto the DuPont dry film will chemically react with the developing solution and be washed away by the developing solution, so the electrode concave mold was made. Rinse the developed ITO glass with deionization, dry it with nitrogen, and bake it in constant temperature oven for 5min.

(e) **Filling**: First, PDMS and curing agent are prepared according to a mass ratio of 10:1, stirred evenly and placed in a vacuum tank (280A Fisher Scientific) for 20 min to remove bubbles. Then, Ag powder and PDMS were mixed uniformly according to the weight ratio of 15:85, and then left at room temperature for 30 min, so the AgPDMS glue was made. Fill the AgPDMS glue into the electrode concave mold, press it tightly, and then put it in a 150℃ constant temperature oven to bake to cure the AgPDMS glue.

(f) **Demembrane**: Firstly, take out the glass pieces in the oven, put them into 5wt% NaOH solution for 5 min after cooling, and removed the redundant DuPont dry film. Then rinse with deionized water, dry with nitrogen and bake in constant temperature oven.Through the above process, 3D microelectrode structure is made. As shown in Figure S3b, Figure S3b is an enlarged view of the 3D microelectrode structure under the microscope.

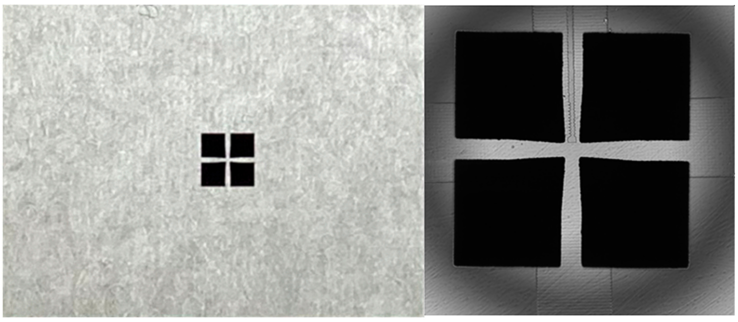

Figure S3. Microelectrode figure (**a**) The mask of microelectrode (**b**) The micrograph of microelectrode

Research shows[1], the threshold concentration for the onset of good conductivity in AgPDMS composites is about 83 wt%. The conductivity r is seen to increase rapidly beyond the threshold, as shown in Figure S4.

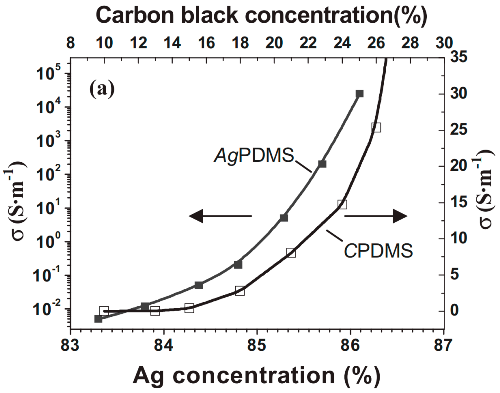

Figure S4. Conductivity versus powder weight concentration

4. The fabrication of PDMS channel layer

Before making microchannel, we need to design the channel graph through AutoCAD software, and then make the mask, The mask of PDMS channel was shown in Figure S5a. The white light-transmitting area is the pattern of the PDMS channel. The production of the PDMS channel layer is still using photolithography technology to transfer the designed mask pattern onto the DuPont dry film through ultraviolet exposure. Since the DuPont dry film is a negative photoresist, the channel produced is a punch. The thickness of DuPont dry film is 38 μm, so the height of PDMS channel processed is also 38 μm. In this way, the height of the channel is the same as that of the microelectrode, and the tightness can be guaranteed.

The fabrication process of PDMS channel includes 6 steps: cleaning, laminating, exposure,

development, pouring and curing, the first 4 steps are the same as before, after the 4 steps, the base mold of the channel was completed, the photograph of the finished channel base mold was shown in Figure S5b. The purpose of the other two steps is to make the PDMS channel layer.

(a) **Pouring**: First of all, PDMS and curing agent are prepared according to the mass ratio of 10:1. After stirring evenly, it is placed in a vacuum tank for 20 min to remove bubbles. Then pour it on the developed punch mold.

(b) **Curing**: Place the poured glass base in a constant temperature oven and bake for 2 hours to cure the PDMS. Then, carefully remove the cured PDMS channel from the base mold, and the channel is completed.

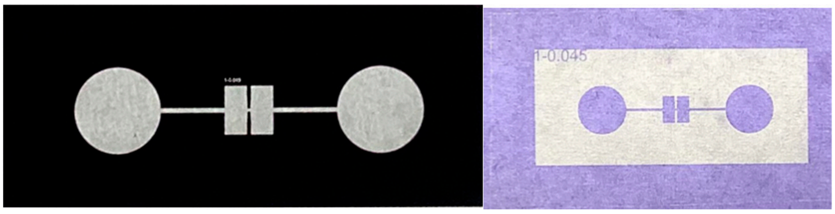

Figure S5. (**a**) The mask of PDMS channel (**b**) The photograph of the finished channel base mold

5. The bonding of DEP separation chip

Through the above steps, the ITO substrate, the 3D microelectrode layer and the PDMS channel layer of the DEP separation chip were fabricated. This step needs to package the above three layers into a complete chip. The steps of bonding are as follows:

(a) **Punching**: First, trim the channel to a suitable size, and then use a puncher to punch holes at the inlets and outlets of the channel. Then blow the debris in the hole clean with nitrogen.

(b) **Plasma cleaning**: Place the ITO substrate with 3D microelectrode and PDMS channel in the plasma for 2 min to increase the hydrophilicity of the surface.

(c) **Packaging**: First, place the 3D microelectrode under the microscope, adjust the microscope focal length, and accurately encapsulate the PDMS channel and the 3D microelectrode.

Through the above steps, the DEP separation chip is completed. The completed DEP separation chip is shown in Figure S6.

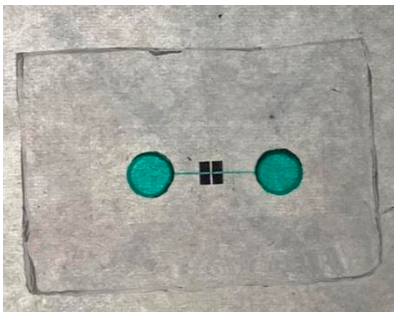

Figure S6. The physical picture of DEP separation chip

Reference

[1] Niu X , Peng S , Liu L , et al. Characterizing and patterning of PDMS-based conducting composites[J]. Advanced Materials, 2007(18).
